# Recent Advances in Supramolecular Systems for Precision Medicine: Structural Design, Functional Integration, and Clinical Translation Challenges

**DOI:** 10.3390/pharmaceutics17091192

**Published:** 2025-09-13

**Authors:** Xiaomin Ma, Yazhe Xiao, Shuyu Li, Jianghai Du, Junjie Wang, Xingzhou Peng

**Affiliations:** 1 State Key Laboratory of Digital Medical Engineering, Key Laboratory of Biomedical Engineering of Hainan Province, School of Biomedical Engineering, Hainan University, Sanya 572025, China; maxiaomin@hainanu.edu.cn (X.M.); lishuyu@hainanu.edu.cn (S.L.); dujianghai@hainanu.edu.cn (J.D.); 2 NHC Key Laboratory of Tropical Disease Control, School of Life Science and Medical Technology, Hainan Medical University, Haikou 571199, China

**Keywords:** supramolecular systems, precision medicine, dynamic covalent bonds, host–guest interactions, targeted drug delivery, bioimaging, clinical translation, biomimetic engineering, stimuli-responsive materials, personalized therapy

## Abstract

Non-covalent and dynamic covalent interactions enable supramolecular systems to function as adaptive platforms in biomedical research, offering novel strategies for precision medicine applications. This review examines five-year developments in supramolecular applications across precision medical domains, including disease diagnosis, bioimaging, targeted drug delivery, tissue engineering, and gene therapy. The review begins by systematically categorizing supramolecular structures into dynamic covalent systems (e.g., disulfide bonds, boronate esters, and hydrazone bonds) and dynamic non-covalent systems (e.g., host–guest interactions, hydrogen-bond networks, metal coordination, and π–π stacking), highlighting current strategies employed to optimize their responsiveness, stability, and targeting efficiency. Representative case studies, such as cyclodextrin-based nanocarriers and metal–organic frameworks (MOFs), are thoroughly analyzed to illustrate how supramolecular systems can enhance precision in drug delivery and improve biocompatibility. Furthermore, this article critically discusses major challenges faced during clinical translation, encompassing structural instability, inadequate specificity of environmental responsiveness, pharmacokinetic and toxicity concerns, and difficulties in scalable manufacturing. Potential future directions to overcome these barriers are proposed, emphasizing biomimetic interface engineering and dynamic crosslinking strategies. Collectively, the continued evolution in structural optimization and functional integration within supramolecular systems holds great promise for achieving personalized diagnostic and therapeutic platforms, thereby accelerating their translation into clinical practice and profoundly shaping the future landscape of precision medicine.

## 1. Introduction

The foundational work in supramolecular chemistry, pioneered by Jean-Marie Lehn in the 1980s and recognized with the Nobel Prize, shifted the research paradigm from isolated molecular properties to functional systems governed by intermolecular interactions [1]. These systems assemble via dynamic self-organization driven by non-covalent forces—such as hydrogen bonding, metal coordination, and π–π stacking (with bond energies typically ranging from 4 to 21 kJ mol^−1^)—to form hierarchically ordered architectures [2,3,4]. Their inherent reversibility, spontaneous organization, and responsiveness to environmental stimuli establish supramolecular systems as transformative platforms for biomedical research [5,6].

Conventional medical approaches face significant limitations in treating complex diseases, particularly cancers and chronic disorders. Challenges include systemic toxicity from non-specific chemotherapeutic agents, inefficient delivery of gene therapy vectors, and the inability of traditional tissue scaffolds to replicate the dynamic extracellular matrix (ECM) microenvironment [7,8,9]. These limitations not only compromise therapeutic efficacy but also pose additional health risks. The emergence of precision medicine addresses these issues by aiming for molecular-level disease diagnosis and intervention to maximize treatment outcomes while minimizing adverse effects. Within this framework, intelligent material platforms capable of sensing physiological cues and executing controlled functions become critically important [10,11].

Supramolecular systems provide powerful impetus for precision medicine through their unique dynamic properties and molecular recognition capabilities. Relying on relatively weak non-covalent interactions, supramolecular assemblies exhibit high sensitivity to physiological triggers, including pH gradients, temperature fluctuations, light irradiation, and specific enzyme activation [12,13]. This stimuli-responsiveness enables precise spatiotemporal control over drug release kinetics, optimization of imaging signal output, and dynamic modulation of material mechanics in vivo. For instance, β-cyclodextrin-based probes functionalized with adamantane-modified cRGD achieve rapid and stable imaging signals [14], while zinc chloride-coordinated self-assembled nanoparticles demonstrate pH-dependent activation, significantly enhancing antitumor efficacy in photodynamic therapy (PDT) [15].

A central challenge in precision medicine lies in achieving molecularly targeted recognition and regulation. Supramolecular systems address this through programmable non-covalent interactions—such as host–guest recognition, hydrogen-bond networks, and π–π stacking [4,16,17]. These interactions facilitate precise delivery of therapeutic agents (e.g., γ-cyclodextrin-encapsulated regorafenib for reprogramming tumor microenvironments [14]), imaging probes (e.g., multimodal agents integrating catalytic, fluorescent, and magnetic resonance imaging via hydrogen bonding and glycosidic interactions [18]), and gene-editing tools to pathological sites while minimizing off-target effects on healthy tissues [19,20]. Critically, the inherent dynamic compatibility of supramolecular interactions enables the integration of diagnostic, therapeutic, and monitoring functionalities within a single platform, advancing the “theranostics” strategy [21,22].

Thus, supramolecular systems serve as pivotal bridges connecting fundamental molecular science with clinical biomedicine. Their dynamic adaptability, precise molecular recognition, and multifunctional integration are reshaping the theoretical foundations of drug delivery, diagnostics, and tissue regeneration, accelerating the realization of personalized precision medicine [23]. Ongoing interdisciplinary research continues to reveal their considerable potential in addressing intractable diseases such as cancer and neurodegenerative disorders [24].

Nevertheless, translating laboratory breakthroughs into clinical practice remains challenging. The clinical translation of supramolecular systems confronts substantial hurdles. Biocompatibility concerns persist, including potential bioaccumulation or toxicity of components (e.g., rare-earth elements, specific organic ligands) [25]. Biological complexity further compromises performance, as protein corona formation can obscure targeting ligands and reduce specificity [26]. Additionally, intricate synthesis routes—such as multi-step pillararene synthesis or functionalization with acrylamide groups—present obstacles to scalable manufacturing, batch-to-batch consistency, and cost-effectiveness [27]. Long-term structural stability under physiological conditions, precise discrimination between pathological signals (e.g., tumor versus inflamed microenvironments), comprehensive pharmacokinetic profiling, and toxicity assessments constitute further critical barriers requiring resolution [28].

**Figure 1:**
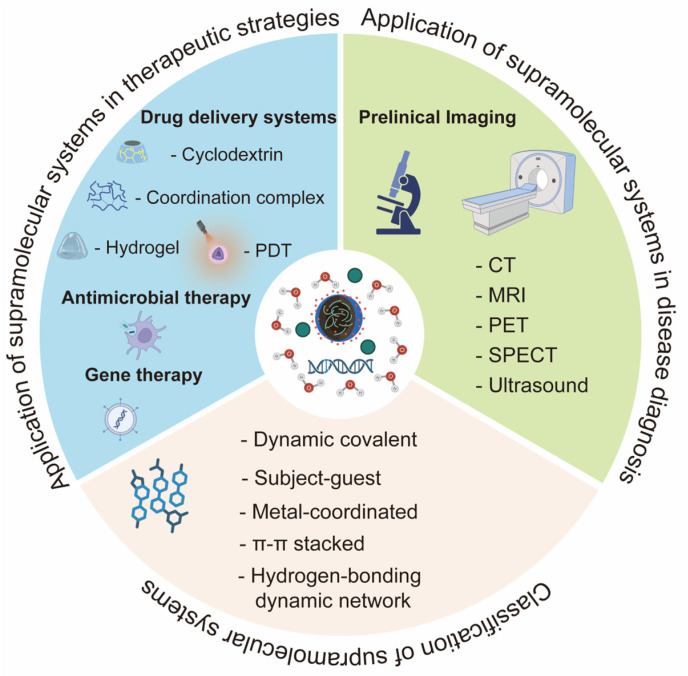
Structural classification and biomedical applications of supramolecular systems.

This review focuses on the latest representative advances of supramolecular systems in driving precision medicine. The content will center on their structural design principles (dynamic covalent assemblies and non-covalent assemblies; see Figure 1), with emphasis on exploring innovative mechanisms and cutting-edge functional designs in core biomedical directions, including disease diagnosis, biological imaging, intelligent drug delivery (covering chemotherapy, gene therapy, and antimicrobial applications), and tissue engineering while providing a systematic comparison of major supramolecular architectures and their biomedical applications (Table 1). Representative cases include cyclodextrin-based nanocarriers for co-delivery of genes and chemotherapeutic drugs to overcome drug resistance [29], high-performance tumor imaging probes constructed using cucurbiturils [30,31], host–guest complexes for delivering regorafenib through γ-cyclodextrin to improve colorectal cancer treatment [14], and responsive nanomedicines formed by metal coordination self-assembly [32,33], among others. Building upon these developments, the review prioritizes overcoming clinical translation barriers: biocompatibility, targeting efficiency, scalable manufacturing, stability, and specificity. Through critical examination of root causes and solution pathways, it maps future trajectories for implementing supramolecular technologies in precision healthcare.

## 2. Classification and Design Strategies for Supramolecular Systems

**Figure 2:**
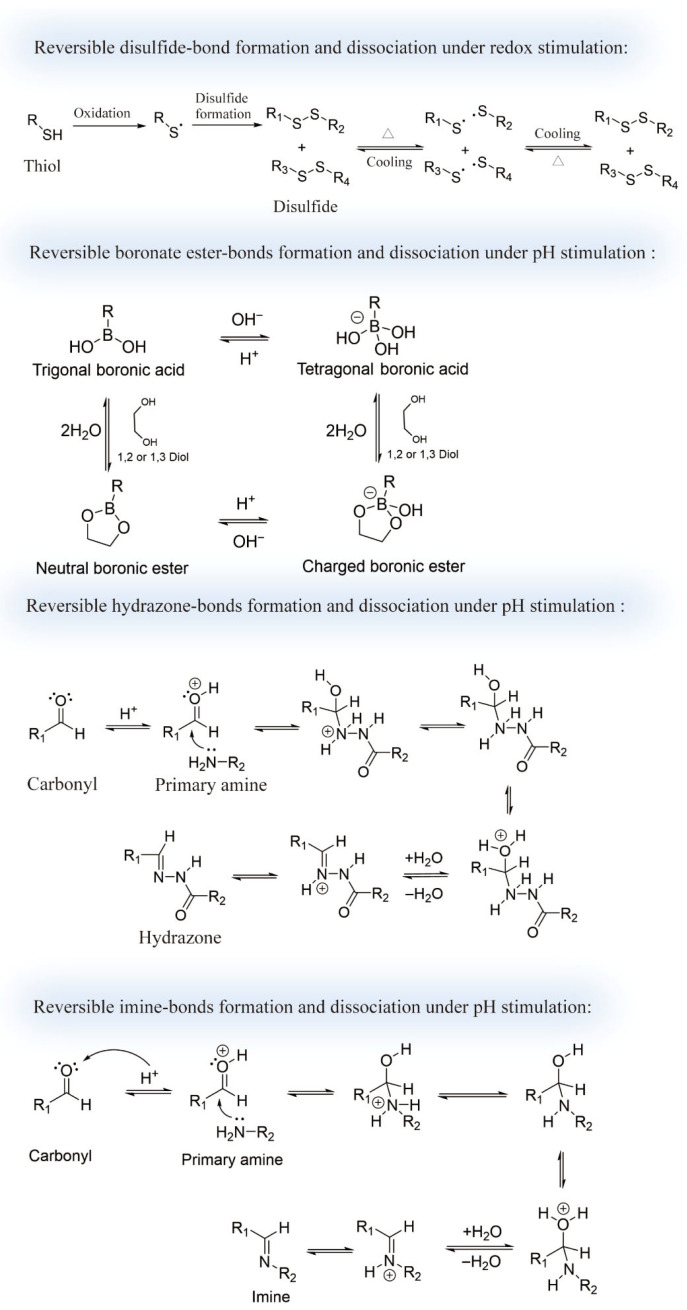
Representative dynamic covalent motifs in supramolecular systems.

Biomedical supramolecular systems require classification based on both functional roles and underlying interaction mechanisms. With the increasing integration of supramolecular chemistry and biomedical science, these systems can be broadly divided into two principal categories: dynamic covalent supramolecular systems and dynamic non-covalent supramolecular systems. Such classification encompasses bonding mechanisms (covalent versus non-covalent) alongside biomedical functional requirements, such as stimuli-responsiveness, targeting specificity, and biocompatibility. Within the dynamic non-covalent category, further subclassifications include host–guest complexes, hydrogen-bonding network systems, metal-coordinated assemblies, and π–π stacked architectures, collectively spanning the continuum from molecular design to clinical translation. Dynamic covalent systems are constructed via reversible covalent bonds (e.g., disulfide, boronate ester, and hydrazone linkages), which allow the formation of stimuli-responsive structures tailored to physiological conditions [77,78,79] (Figure 2). These systems are particularly suited for controlled drug release, tissue engineering, and biosensing, owing to their balance between stability and environmental sensitivity [80]. In contrast, dynamic non-covalent assemblies rely on weaker, reversible interactions—such as hydrogen bonding, metal coordination, host–guest recognition, and π–π stacking—offering advantages in reversibility, molecular selectivity, and multi-component compatibility, and have therefore become a predominant strategy in biomedical supramolecular design [81].

### 2.1. Dynamic Covalent Supramolecular Systems

Reversible covalent linkages enable dynamic systems to serve as degradable drug carriers and tissue engineering scaffolds. A representative example involves disulfide-based constructions, which are responsive to redox conditions. In such systems, thiol-containing molecules (e.g., cysteine or thiolated PEG) undergo disulfide bond cleavage or formation in response to environmental redox stimuli [34]. Sun and co-workers showed doxorubicin-loaded lipid nanoparticles with disulfide functionalization maintain extracellular stability while degrading rapidly in reductive tumor cell environments, resulting in a threefold enhancement in intracellular drug release compared to non-responsive carriers [35]. In pancreatic cancer models, disulfide-based delivery systems have been shown to selectively accumulate and release drugs in glutathione (GSH)-rich tumor regions, substantially reducing off-target toxicity [36].

Other representative systems include boronate ester and hydrazone linkages, which exhibit sensitivity to pH variations in physiological environments. Boronate esters (B–O–C bonds) hydrolyze under mildly acidic conditions (pH ≈ 6.5), making them particularly suitable for inflammation- or tumor-targeted applications [37]. For instance, Zhang and colleagues engineered cyclodextrin-based nanoparticles modified with boronate esters for co-delivery of siRNA and chemotherapeutic agents; under the acidic tumor milieu, these carriers released their payloads efficiently, enhancing the tumor suppression rate by over 40% in breast cancer models [38]. Hydrazone bonds (C=N–N), another acid-sensitive motif, also undergo reversible cleavage under acidic conditions; however, pharmacokinetic analyses indicate a plasma half-life of approximately 5 h at physiological pH (7.4) [39].

Despite the benefits of structural responsiveness and tunability, dynamic covalent systems face notable limitations. From a bond energy perspective, strong covalent linkages impart high systemic stability but require harsh triggers—such as elevated temperature or low pH—for bond cleavage, which exceed physiological tolerances and limit in vivo applicability. Conversely, weaker covalent bonds (e.g., acylhydrazones) are more responsive but prone to premature cleavage in circulation, leading to untimely drug release [40]. From a microenvironmental standpoint, the acidic pH of tumor tissues (≈6.5) overlaps with that of inflamed tissues (≈6.8–7.0) and intracellular compartments such as lysosomes (≈4.5–5.5), complicating selective activation. This ambiguity can result in off-target drug release in non-malignant inflammatory sites, particularly in hydrazone- or boronate-containing systems [41]. Fundamentally, these challenges reflect an inherent tension between dynamic responsiveness and physiological stability [42]. Overcoming this conflict will require the rational molecular design of chemical linkages that are finely attuned to the physicochemical constraints of the intended biomedical application, thus achieving an optimal balance between functionality and biocompatibility.

### 2.2. Dynamic Non-Covalent Supramolecular Systems

Dynamic non-covalent supramolecular systems have emerged as a central focus in biomedical supramolecular research, owing to their unique reliance on weak, reversible interactions such as hydrogen bonding, metal coordination, host–guest recognition, and π–π stacking, typically characterized by bond energies in the range of 4–21 kJ mol^−1^ [81]. These weak forces endow the system with dynamic reversibility, enabling the supramolecular architecture to respond to physiological cues in real time by adjusting its structure—an intrinsic feature that differentiates it from static covalent counterparts [4,5]. Furthermore, the directional nature and spatial complementarity of non-covalent interactions allow for precise molecular recognition at the nanoscale. The compatibility of multiple weak interactions within a single framework also enables the concurrent integration of diverse functional modules—therapeutic agents, imaging probes, genetic cargos, and catalytic sites—thus allowing coordinated diagnostic, therapeutic, and monitoring functionalities [18]. Collectively, these properties confer significant advantages over traditional covalent systems in fields such as drug delivery, bioimaging, and tissue engineering.

#### 2.2.1. Host–Guest Supramolecular Systems

Host–guest recognition represents one of the fundamental principles of supramolecular chemistry, wherein selective binding is achieved between a host and a guest molecule through structural complementarity (in terms of size and geometry) and energy compatibility (via non-covalent interactions such as hydrogen bonding, hydrophobic effects, π–π stacking, and electrostatic forces) [11,12]. This dynamic and reversible interaction underpins both supramolecular self-assembly and biological molecular recognition and has shaped research paradigms in biomedicine and materials science [11].

**Figure 3:**
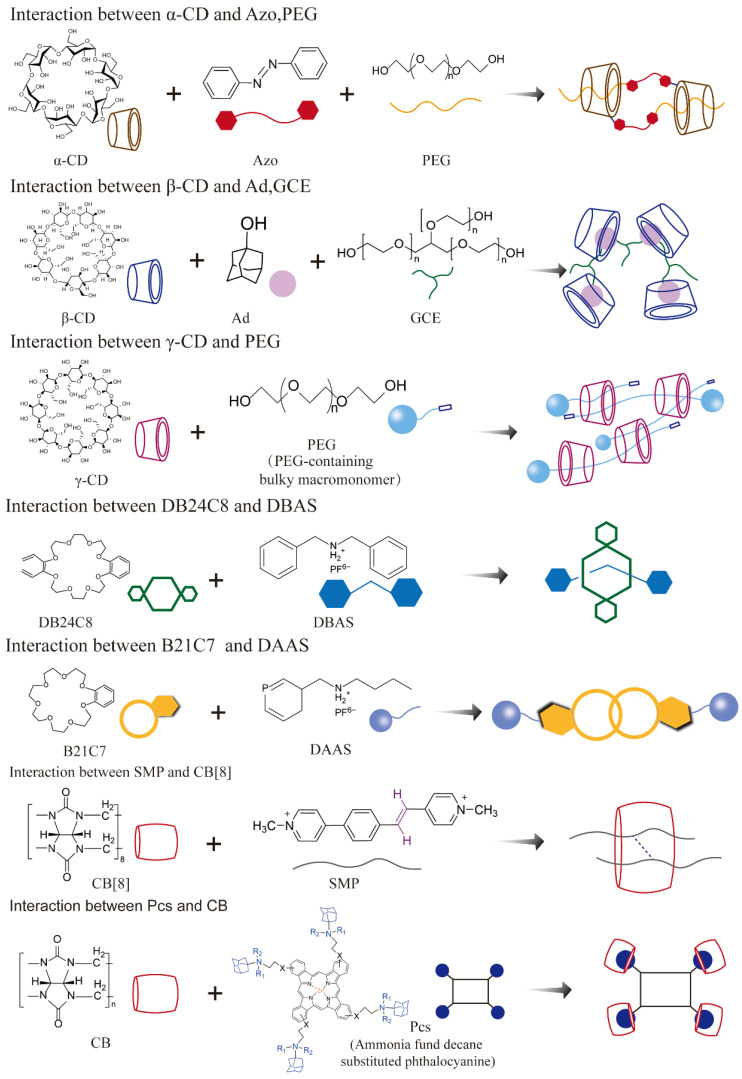
Representative structures of host and guest components in supramolecular systems.

Representative host molecules—including crown ethers, cyclodextrins [20], cucurbiturils, and pillararenes—offer cavities with varying geometries and polarity profiles, enabling selective encapsulation of target guests (Figure 3) [12,17,20]. For example, the hydrophobic interior of cyclodextrins enhances the aqueous solubility of hydrophobic drugs through inclusion complexation [43]; cucurbiturils, with their rigid cavities, preferentially bind cationic species via ion–dipole interactions; and pillararenes, regarded as the fifth-generation macrocyclic hosts after crown ethers, cyclodextrins, calixarenes, and cucurbiturils, possess symmetrical and rigid architectures with multivalent binding sites, which facilitate the construction of multifunctional supramolecular assemblies [44,45]. Mechanically interlocked molecules (MIMs), such as rotaxanes and polyrotaxanes, exemplify host–guest systems with dynamic mechanical bonds that are sensitive to environmental stimuli. For instance, pH-responsive rotaxane-based doxorubicin carriers have been shown to enhance chemotherapeutic efficacy by triggering drug release specifically in the acidic tumor microenvironment [46].

Host–guest recognition is also widely observed in biological systems. Antigen–antibody interactions involve complementarity between the Fab region of antibodies and specific epitopes through hydrogen-bonding networks and hydrophobic contacts; transcription factors insert α-helices into the major groove of DNA and achieve sequence specificity via hydrogen bonding (e.g., adenine-N^6^ to glutamine) and electrostatic interactions (e.g., arginine–phosphate pairing); enzyme active sites undergo conformational adjustments to accommodate substrates—paralleling a dynamic version of the lock-and-key model—to enhance catalytic efficiency. These natural systems offer crucial design insights for biomimetic supramolecular carriers [12,13].

Despite their dynamic responsiveness and modularity, host–guest systems face limitations in targeted delivery and drug loading, necessitating further chemical optimization. For example, once introduced into the bloodstream, supramolecular nanoparticles rapidly adsorb serum proteins to form a “protein corona”, which can obscure targeting ligands or alter binding specificity. Additionally, the constrained cavity size and polarity of macrocyclic hosts impose inherent restrictions on the range and type of guest molecules that can be encapsulated.

#### 2.2.2. Dynamic Systems Based on Hydrogen-Bonding Networks

Hydrogen-bonding networks are generally classified into three types based on their structural complexity and interaction modes: (1) primary networks rely on single, reversible hydrogen bonds, offering directionality and dynamic reversibility; (2) dual cross-linked networks integrate multiple hydrogen bonding motifs or combine hydrogen bonds with other reversible interactions, such as covalent hybrids or metal coordination, to enhance structural stability and responsiveness; and (3) multivalent cross-linked networks involve cooperative interactions among multiple dynamic bonds, often including triple or quadruple hydrogen bonding arrays. These densely packed networks provide high binding affinity, mechanical robustness, and excellent self-healing capabilities, making them suitable for applications such as biomimetic scaffolds, supramolecular adhesives, and stimuli-responsive hydrogels [47].

**Figure 4:**
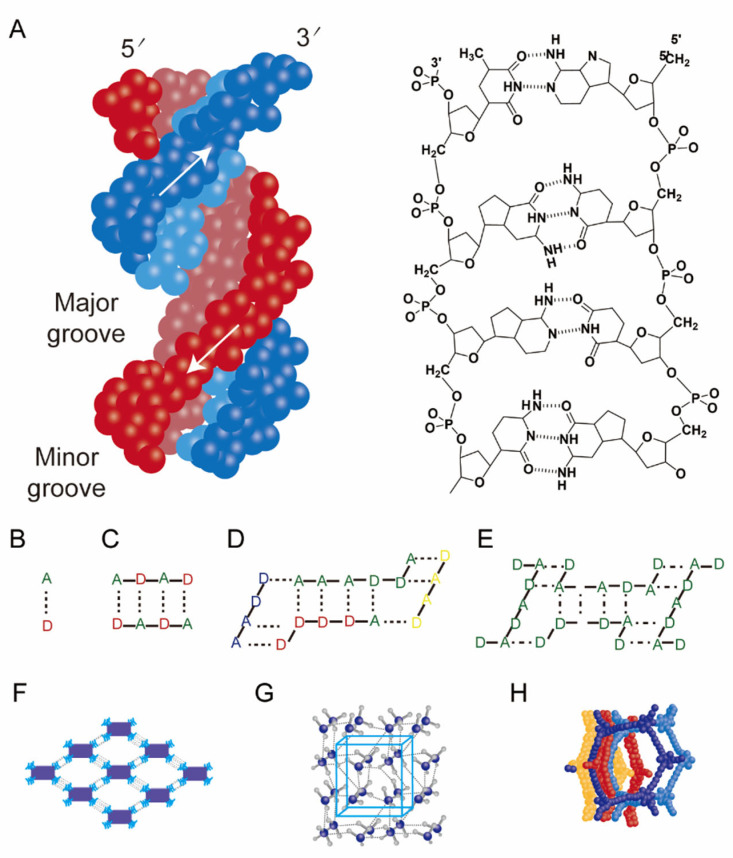
Representative types of hydrogen-bond-assembled supramolecular systems. (**A**) Naturally occurring hydrogen bonds. (**B**,**C**) Hydrogen bonding between individual molecules. “D”: proton donor; “A”: proton acceptor; dashed lines represent hydrogen bonds; solid lines represent covalent bonds, different colors represent different molecules. (**D**) Hydrogen bonding between different molecules. (**E**) Quadruple hydrogen bonding between identical molecules. (**F**–**H**) Various hydrogen-bonded framework structures.

Primary hydrogen-bond networks are built upon electrostatic attractions between electronegative atoms (e.g., O, N, and F) and hydrogen atoms, resulting in dynamic or static interaction matrices. With interaction strengths ranging from 1 to 40 kJ mol^−1^—intermediate between van der Waals forces and covalent bonds—these networks exhibit directionality, saturability, and reversibility. The density and pattern of hydrogen bonds modulate physicochemical properties such as melting point, solubility, and mechanical strength, as well as biological functions, including the structural integrity of DNA helices (Figure 4A). These systems have found practical relevance in biomedical materials such as cartilage-repair scaffolds and injectable hemostatic fluids [48].

Dual cross-linked networks integrate hydrogen bonding with additional reversible interactions to achieve enhanced stability and environmental responsiveness. A representative system is carboxyl–amine hydrogen-bonded supramolecular assemblies, arising from electrostatic and orbital hybridization between carboxyl oxygen atoms and hydrogen donors in amino groups (–NH_2_/–NH–). These interactions undergo proton-mediated reversible association—stable at neutral pH and cleavable under acidic conditions [77,78]—making them well-suited for stimuli-responsive drug release in tumor or inflamed microenvironments, as well as for mucosal adhesion applications (Figure 4B–E). Another example involves hydrogen-bonded organic frameworks (HOFs), crystalline porous materials constructed via hydrogen bonding and other non-covalent interactions. Characterized by high porosity, tunable pore size, and large surface area, HOFs are regarded as promising delivery carriers [49]. Their synthetic flexibility—often enabling self-assembly in aqueous environments—permits integration with enzyme-sensitive or biologically active components. For instance, Pan and colleagues developed enzyme-catalyzed HOFs for in situ, light-triggered CRISPR/Cas9 gene editing, achieving effective gene regulation and tumor inhibition in hepatocellular carcinoma models [50,51].

Multivalent hydrogen-bonding networks are molecular architectures formed through directional hydrogen bonds between complementary donor and acceptor groups, establishing highly specific and reversible interactions that enhance binding affinity and structural stability, as demonstrated in quadruple hydrogen-bonding systems like ureido-pyrimidinone (UPy) dimer [81]. In supramolecular polymers, these networks leverage repetitive binding motifs to form densely packed hydrogen-bond arrays (Figure 4F–H) [52], conferring exceptional dynamic reversibility and self-healing capabilities. Such polymers—often incorporating high-affinity moieties like UPy—exhibit mechanical robustness, stimuli responsiveness, and signal transduction functionality, rendering them suitable for load-bearing tissue engineering scaffolds that replicate extracellular matrix (ECM) mechanics and wearable biosensors detecting biomechanical signals [53]. Furthermore, in peptide-based hydrogels, multivalent hydrogen bonding facilitates mechanotransduction pathways that guide cell differentiation and enhance regenerative outcomes.

Nonetheless, the application of hydrogen-bond-driven supramolecular systems is constrained by intrinsic limitations and physiological complexities [54]. The dynamic reversibility of hydrogen bonds can undermine structural stability—for example, carboxyl–amine networks tend to dissociate prematurely under acidic inflammatory conditions [55], and batch-to-batch inconsistency remains a major hurdle in scalable HOF production due to structural heterogeneity [56]. Moreover, certain modified frameworks exhibit risks of bioaccumulation or potential toxicity, especially when metal components are involved [57]. Despite these challenges, advances in molecular engineering—such as the integration of dynamic covalent–hydrogen bond hybrids—have substantially improved system stability and biosafety. Future research should prioritize elucidating structure–property relationships under physiological conditions, resolving fabrication scalability, and ultimately balancing functionality with clinical safety.

Building upon strategies to enhance system stability (e.g., dynamic covalent–hydrogen bond hybrids introduced in [58]), hydrogen bond triple cross-linked networks emerge as an advanced design that synergistically leverages multiple dynamic bonds—such as ionic interactions, hydrogen bonds (both synergistic and anti-synergistic types), and dynamic disulfide bonds [58]. However, the structural complexity of these networks demands precise synthesis techniques for practical implementation, a challenge particularly relevant to hydrogel and supramolecular hydrogel materials [47]. Exemplifying this approach, Zhang’s team utilized imidazolidinyl urea (IU)—rich in hydrogen bond donors/acceptors—as an enhancer. By copolymerizing IU with hydrophilic PEG-based polyurethane (PEG-PU) and hydrophobic ε-caprolactone-based polyurethane (PCL-PU), they engineered multiple hydrogen bonds between phases. This critical intervention prevented PCL-PU uneven aggregation, thereby inducing a uniform bicontinuous phase structure [59].

#### 2.2.3. Metal-Coordinated Supramolecular Systems

**Figure 5:**
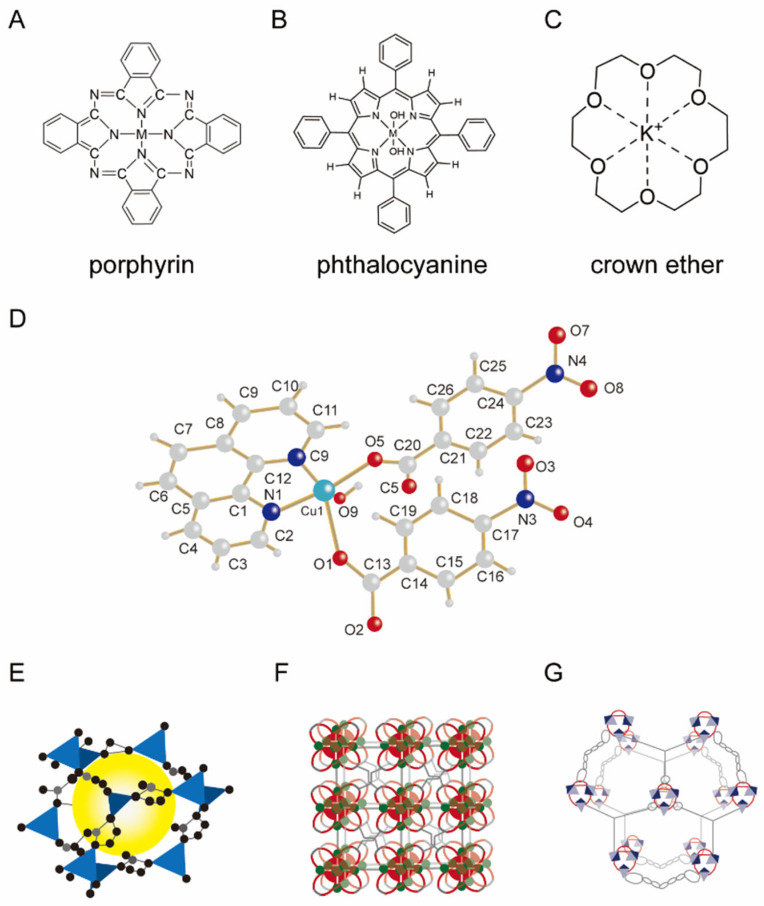
Metal-coordinated supramolecular systems. (**A**) Coordination between metal ions and porphyrin molecules; (**B**) coordination between metal ions and phthalocyanine molecules; (**C**) crown ether–metal coordination; (**D**) chain-like structures formed by metal–ligand complexes; and (**E**–**G**) metal–organic frameworks (MOFs) formed by metal ion coordination.

Metal-coordinated supramolecular systems are dynamic functional assemblies formed through coordination interactions between metal ions and organic ligands (Figure 5). These systems harness the directionality, tunability, and reversibility of coordination bonds to integrate the optical, magnetic, and catalytic properties of metal centers with the structural diversity of organic ligands, enabling the construction of functionally precise architectures for biomedical applications [22]. Their key advantages include stimulus-responsive behavior—triggered by pH, light, or enzymatic cues—multifunctional integration (e.g., theranostics), and structural programmability for encapsulating a wide range of therapeutic agents [13]. Such systems have been widely investigated for targeted drug delivery, phototherapy, and diagnostic imaging, including MRI and fluorescence-based techniques.

Metal ions commonly employed in coordination assemblies can be broadly classified into three categories: transition metals, rare-earth metals, and main-group metals. Transition metals (e.g., Fe^2^⁺/Fe^3^⁺, Cu^2^⁺, and Pt^2^⁺) possess unpaired d-electrons and adopt diverse coordination geometries (such as octahedral or square planar), making them particularly suited for constructing responsive and catalytically active supramolecular platforms [25,60]. Coordination strength can be modulated through ligand field effects and chelating ligands (e.g., bipyridine and thiol) or by introducing stimuli-responsive moieties (e.g., pH-sensitive carboxylates), allowing responsiveness to tumor microenvironmental conditions such as acidity, high glutathione levels, or specific enzyme expression. Representative applications include Fe^2^⁺/Fe^3^⁺–tannic acid coordination polymers that generate reactive oxygen species via the Fenton reaction to induce apoptosis in drug-resistant tumor cells [60]; Pt^2+^ prodrugs encapsulated in metal–organic frameworks (MOFs) that remain stable during circulation and selectively release cytotoxic agents at tumor sites [61]; and Cu^2^⁺–bipyridine-based elastomers that exhibit self-healing properties through reversible bond exchange, suitable for wearable sensors and damage-responsive materials [62].

Rare-earth metals (e.g., Gd^3+^, Eu^3+^, and Yb^3+^), which exploit 4f electron transitions, offer unique advantages for optical imaging (e.g., fluorescence and upconversion luminescence) and magnetic resonance diagnostics. Their coordination with rigid macrocyclic ligands such as DOTA or β-diketones enhances both complex stability and signal sensitivity [25].

Main-group metals (e.g., Mg^2+^, Ca^2+^, and Al^3+^), owing to their essential biological roles and relatively low toxicity—such as Mg^2+^ in ATP coenzymes or Ca^2+^ in bone matrix—represent ideal candidates for constructing biocompatible materials. Coordination with biologically derived ligands such as phosphate or amino acids can yield low-coordinate structures under mild conditions (neutral pH and ambient temperature), with degradation products that align with natural metabolic pathways. These systems are particularly valuable in mimicking biomineralization processes (e.g., hydroxyapatite formation) and facilitating molecular delivery in tissue repair applications [63].

Despite their promise, metal-coordinated supramolecular systems face several inherent challenges rooted in metal-specific properties and coordination chemistry. For transition metals, concerns include potential cytotoxicity from free metal ions (e.g., Pt^2+^ and Cu^2+^), instability under physiological conditions, premature or delayed responses to stimuli, and reduced therapeutic efficacy due to antioxidant mechanisms within tumors that attenuate Fenton-based ROS generation [64,65]. Rare-earth-based systems may suffer from long-term accumulation in organs (e.g., Gd^3+^ retention in the liver and spleen), susceptibility to fluorescence quenching by serum proteins, and complex synthetic procedures that hinder scalability [25]. Main-group metal systems often exhibit insufficient coordination stability (e.g., hydrolysis of Ca^2+^–phosphate bonds), limited responsiveness (predominantly relying on endogenous cues), and restricted functional diversity due to electronic structure constraints [63].

## 3. Current Research Progress

### 3.1. Applications of Supramolecular Systems in Disease Diagnosis

In the field of bioimaging, supramolecular biomaterials have demonstrated unique advantages in visualizing biological processes at the molecular level. By incorporating imaging agents into their architectures, these systems can significantly enhance the contrast and resolution of various imaging modalities, including magnetic resonance imaging (MRI), fluorescence imaging, and positron emission tomography (PET) [46]. Furthermore, their ability to selectively target specific biomolecules or cellular structures has accelerated the development of advanced imaging probes for early disease detection [82]. The diagnostic performance of such probes largely depends on three critical attributes: structural stability, targeting specificity, and signal sensitivity—all of which are essential for accurate visualization of molecular signatures within cells, tissues, or organs. Conventional probes, including radiolabeled agents, fluorescent tracers, and ultrasound-responsive nanomaterials, often suffer from limited in vivo stability, off-target interactions, short imaging windows, and potential toxicity, thereby restricting their clinical applicability [83].

**Figure 6:**
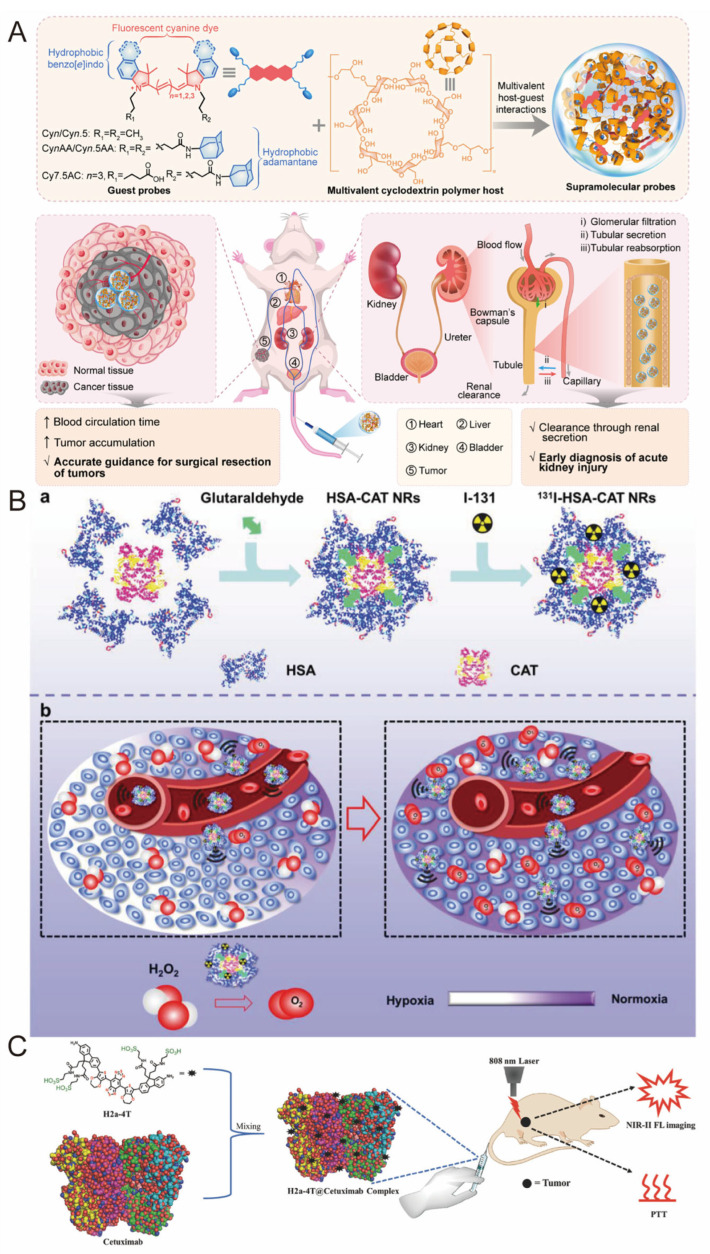
(**A**) Schematic illustration of a multivalent supramolecular fluorescent probe for precise disease imaging [84]. Copyright 2024, The Royal Society of Chemistry. (**B**) Schematic illustration of a hybrid protein nanoreactor (131I–HSA–CAT NRs) designed for simultaneous oxygen generation and iodine-131 delivery to enhance radionuclide therapy. (**a**) HSA-CAT NRs exhibit a high labeling efficiency of ^14^I, which is attributed to their rich intrinsic tyrosine residues. (**b**) By recording the signals of the fluorescent label and ^15^I, it was demonstrated that after intravenous injection of ^15^I-HSA-CAT NRs, there is an efficient passive tumor homing ability, significantly alleviating tumor hypoxia through CAT-mediated endogenous H_2_O_8_ decomposition [67]. Copyright 2019 Wiley-VCH. (**C**) Diagram of near-infrared II dye–protein complex for biomedical imaging and imaging-guided photothermal therapy [68]. Copyright 2018 Wiley-VCH.

A major clinical challenge remains the rapid clearance of imaging probes from pathological sites by the immune system, particularly the mononuclear phagocyte system (MPS), which compromises both retention time and imaging efficiency [84]. Optical imaging, while powerful for early diagnosis and treatment planning, is often hindered by MPS uptake that leads to decreased signal accuracy [85]. By introducing multiple hydrophobic groups into the dye structure, the system achieved two principal benefits: improved probe stability through enhanced supramolecular interactions and minimized energy loss, resulting in higher fluorescence efficiency (Figure 6A) [84]. Additionally, precise control over particle size and surface properties via self-assembly strategies enabled evasion of MPS-mediated clearance and prolonged circulation time in vivo [84]. These optimizations collectively improved imaging clarity by reducing background noise and facilitated real-time, high-resolution visualization in models of acute kidney injury and solid tumors, offering valuable guidance for early intervention and image-guided surgery [86].

Abnormal levels of biothiols such as cysteine, homocysteine, and glutathione have been implicated in the pathogenesis of various diseases, including Alzheimer’s disease, Parkinson’s disease, and HIV/AIDS. Given the critical roles of these thiol-containing species in cellular redox homeostasis, the development of real-time sensing platforms for monitoring intracellular biothiol concentrations is essential for elucidating their pathophysiological relevance [30]. Liu and colleagues constructed a supramolecular probe by coupling a coumarin-functionalized β-cyclodextrin (rCP-βCD) with a cRGD-modified adamantane conjugate containing arginine, glycine, and aspartic acid (cRGD-ADA). This host–guest complex enabled cancer-targeted sensing and high-sensitivity detection of biothiols, facilitated through glycosidic and hydrogen-bonding interactions for rapid and stable signal output [66].

Two major classes of supramolecular bioorganic complexes have emerged for targeted bioimaging: protein–dye conjugates and protein-responsive nanomaterials [87]. Yang and co-workers developed a hybrid protein nanoreactor by crosslinking human serum albumin (HSA) and catalase (CAT) using glutaraldehyde and encapsulated iodine-131. This nanoreactor preserved enzymatic stability while allowing effective radiolabeling with the therapeutic radionuclide. Upon co-delivery with hydrogen peroxide and a photosensitizer, the resulting protein–dye hybrid facilitated oxygen generation through H_2_O_2_ decomposition, alleviating tumor hypoxia and enhancing imaging performance (Figure 6B) [67]. In a parallel approach, a water-soluble, biocompatible near-infrared II (NIR-II) fluorophore (H2a-4T) was combined with fetal bovine serum (FBS) and Cetuximab to yield a protein–dye complex with excellent optical properties and targeting specificity. High-resolution, real-time imaging of mouse hindlimb vasculature and lymphatics was achieved using H2a-4T@FBS, and the system demonstrated utility in NIR-II-guided sentinel lymph node surgery (Figure 6C) [68].

To achieve tumor-specific imaging, supramolecular MRI contrast agents have been designed to target overexpressed receptors on cancer cell surfaces, leveraging hydrogen bonding, π–π stacking, and hydrophobic interactions to promote accumulation at pathological sites [88]. To overcome the side effects associated with gadolinium-based agents, Gassensmith and colleagues employed cucurbiturils to form nanomolar-affinity complexes with organic radical contrast agents (ORCAs), shielding them from in vivo reduction. These ORCAs were further conjugated to self-assembling biomacromolecules to enhance signal retention and imaging contrast [31]. Holland’s team constructed a high-resolution imaging probe based on a β-cyclopentane scaffold terminated with axial ligands. Using co-confocal colocalization strategies, the probe enabled precise targeting of kLCell receptors via hydrogen bonding and glycosidic interactions. Notably, the probe integrated catalytic, fluorescent, and magnetic resonance imaging functionalities, expanding its diagnostic utility [18].

### 3.2. Applications of Supramolecular Systems in Therapeutic Strategies

#### 3.2.1. Supramolecular Drug Delivery Systems

In cancer therapy, imaging plays a critical role in delineating tumor location, size, morphology, and spatial relationships with surrounding tissues. When integrated with treatment protocols, it also enables real-time monitoring of drug release and activation kinetics [89]. To meet the demands of precision oncology, supramolecular delivery systems have been engineered to respond to specific features of the tumor microenvironment—such as acidic pH gradients and elevated glutathione concentrations [90]—facilitating spatiotemporally controlled drug release at pathological sites. These stimuli-responsive mechanisms improve intratumoral drug bioavailability while minimizing systemic toxicity [91]. Moreover, modular design strategies allow the incorporation of functional motifs—such as bioactive peptides and enzyme-responsive units—into bioactive scaffolds, enabling coordinated tuning of mechanical properties, enzymatic degradation kinetics, and cell–material interactions at the molecular level [92].

**Figure 7:**
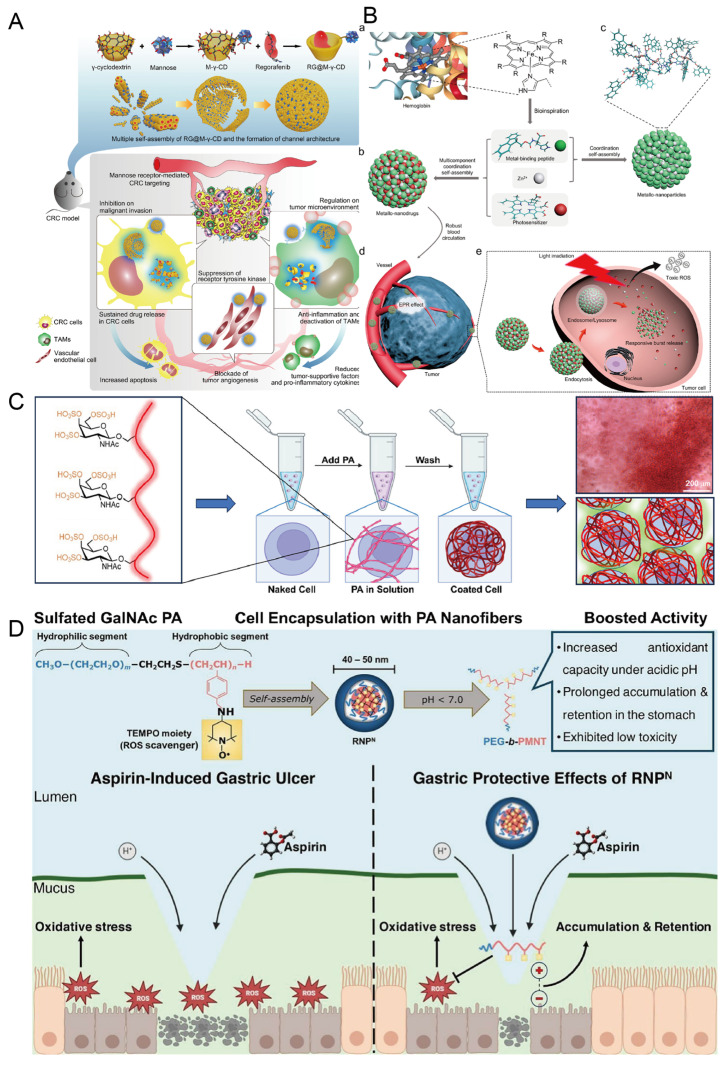
(**A**) Schematic illustration of cyclodextrin-based host–guest complex loading regorafenib for colorectal cancer therapy [14]. Copyright 2021, Springer Nature. (**B**) Schematic illustration of a smart peptide-based supramolecular photodynamic metal nanomedicine constructed via multicomponent coordination-driven self-assembly. (**a**) The heme group in human hemoglobin is biologically coordinated with histidine through metal ions (PDB: 1A3N). (**b**) In the presence of zinc ions, metal nanomedicine is constructed through the synergistic coordination of peptides and photosensitizers. (**c**) A schematic diagram of the molecular organizational pattern of metal-binding peptides and Zn2+. (**d**) Metal nanomedicine accumulates in tumors within a robust blood circulation. (**e**) Metal nanomedicine enters cells via endocytosis, where it undergoes burst release in the cellular compartment environment, activating the released photosensitizers to produce toxic ROS for efficient photodynamic therapy [15]. Copyright 2018, American Chemical Society. (**C**) Schematic illustration of boosting chondrocyte bioactivity with ultra-sulfated glycopeptide supramolecular polymers [71]. Copyright 2024, Elsevier. (**D**) Schematic diagram of the development of oral pH-sensitive redox nanomedicines for gastric ulcer therapy [72]. Copyright 2024, Elsevier.

Cyclodextrins (CDs) represent a key class of supramolecular hosts widely employed in drug delivery owing to their favorable targeting capabilities, structural stability, and biocompatibility, making them highly suitable for clinical translation [93]. Regorafenib (RG), a multikinase inhibitor used in colorectal cancer treatment, functions by blocking the immunoglobulin- and EGF-like domain-containing protein 2 (ILG2) signaling axis, thereby reducing tumor-associated macrophage infiltration [94]. However, the drug suffers from poor solubility and limited membrane permeability [95]. To address these limitations, Bai and colleagues engineered a mannose-modified γ-cyclodextrin (M-γ-CD) capable of encapsulating RG within its hydrophobic cavity, forming the supramolecular complex RG@M-γ-CD. This formulation significantly improved RG’s pharmacokinetic profile, bioavailability, and therapeutic index, while also leveraging the anti-inflammatory properties of cyclodextrin to reprogram the tumor microenvironment (Figure 7A) [14].

Supramolecular coordination complexes represent structurally predictable and size-tunable self-assembled architectures, constructed through directional coordination between metal centers and organic ligands. Their intrinsic hydrophobicity and scalable cavity dimensions facilitate efficient drug encapsulation and transport [96]. In addition, these complexes often exhibit active tumor-targeting capabilities, low systemic toxicity, and enhanced cellular uptake, making them promising candidates for anticancer applications [32]. Ruthenium-based complexes, known for their potent antitumor and antimicrobial activities, suffer from poor aqueous solubility and suboptimal biodistribution under physiological conditions—factors that hinder their clinical translation. To address these limitations, Villemin and colleagues developed several strategies to improve targeted delivery of ruthenium complexes, one of which involves covalent conjugation to polymeric carriers to form prodrugs capable of controlled activation at disease sites [33].

High-output nitric oxide (NO) generated by inducible nitric oxide synthase (iNOS) following inflammatory activation plays a critical role in the pathogenesis of various diseases, particularly septic shock [97]. Fricker and co-workers investigated the NO-scavenging efficacy of potassium chlorido[hydrogen(ethylenediamine)tetraacetate]ruthenate (JM1226), which forms a hydrated complex (JM6245) upon reaction with NO in aqueous solution. Both JM1226 and JM6245 effectively reduced NO accumulation in the culture medium of activated RAW264 macrophages, attenuated vasorelaxation responses to SNAP in isolated rat tail arteries, and protected P815 tumor cells from NO-mediated cytotoxicity by activated immune cells. These findings suggest that ruthenium-based NO scavengers may serve as therapeutic agents to alleviate inflammatory damage associated with septic shock [98].

The integration of nanotechnology with photosensitizers in photodynamic therapy (PDT) has enabled precise tumor ablation while improving the spatial selectivity and reducing off-target toxicity of photosensitizing agents [99]. In particular, supramolecular assemblies with photoresponsive behavior undergo structural transitions under specific light wavelengths, offering tunable electronic and photophysical properties for the development of multifunctional intelligent materials [100]. Yan and colleagues reported the coordination-driven self-assembly of fluorene-methoxycarbonyl-l-histidine (Fmoc-H) and N-benzyloxycarbonyl-l-histidine-phenylalanine (Z-HF) with zinc chloride into nanoparticles with well-defined nanostructures, uniform size distribution, and high photosensitizer loading capacity. These nanoparticles exhibited sharp responsiveness to physiological variations in pH and glutathione levels, prolonged blood circulation times, and significantly enhanced antitumor efficacy under PDT conditions (Figure 7B) [15].

Traditional irreversibly crosslinked hydrogels, while mechanically robust, lack the dynamic properties of the native extracellular matrix (ECM)—a feature essential for tissue regeneration [101]. In vivo, cells continuously interact with and remodel the ECM to orchestrate various cellular behaviors [102]. Reversibly crosslinked hydrogels offer a promising platform to recapitulate such dynamic microenvironments, enabling better modeling of diseased tissues and facilitating studies of cellular function and recovery [103]. Zengin and colleagues developed an ECM-mimicking hydrogel by ultraviolet-initiated thiol–ene crosslinking between norbornene-functionalized benzene-1,3,5-tricarboxamide (NBTA) macromonomers and thiolated mesoporous silica nanoparticles (MSNs). The resulting nanocomposite hydrogel exhibited enhanced mechanical performance and demonstrated excellent cytocompatibility, maintaining the viability of encapsulated MG63 cells in vitro [69]. Further studies have shown that tuning polymer composition and crosslinking strategies enables modulation of the mechanical properties of supramolecular hydrogels; the incorporation of proteins or polymers can mimic biological heterogeneity, while enzymatic or chemical control over crosslinking density and pore architecture enables functional customization of the material [104].

As the body’s largest organ, the skin is particularly susceptible to environmental insults, and severe wounds can substantially impair healing [105]. Silk fibroin (SF)-based wound dressings have shown considerable promise in promoting tissue regeneration [106]. However, conventional SF hydrogels often suffer from slow gelation and poor self-healing capacity [89]. To address these limitations, Yu and co-workers engineered an injectable, self-healing hydrogel by integrating SF, acrylated β-cyclodextrin (Ac-CD), and curcumin (Cur) within a single supramolecular system. This platform demonstrated improved mechanical strength, long-term stability, self-healing behavior, injectability, and biocompatibility [70].

Autologous chondrocyte implantation (ACI)—currently a standard treatment for cartilage degeneration—involves harvesting, expanding, and reimplanting patient-derived chondrocytes. However, ACI remains limited by prolonged recovery, extensive suturing, and potential cell leakage [107,108]. To address these drawbacks, Stupp’s group developed a supramolecular fiber hydrogel derived from oversulfated glycopolymers. This system enhances TGFβ-1 signaling and presents alternating saccharide residues that mimic cartilage-specific ECM architecture, thereby promoting cell migration, proliferation, and secretion of cartilage extracellular matrix proteins. In preclinical models, this hydrogel markedly improved cartilage regeneration outcomes (Figure 7C) [71].

Gastric ulcers are a very common gastrointestinal disease, and their primary pathogenesis is closely associated with oxidative imbalance caused by excessive production of reactive oxygen species (ROS) in the body [109]. In addition, long-term use of low-dose aspirin can also increase the likelihood of complications related to gastric ulcers [110]. Furthermore, when ROS levels are excessive, they can further trigger a series of adverse consequences, such as lipid and protein peroxidation reactions, and may even cause DNA damage [111]. To address these issues related to gastric ulcers, a team of Nagasaki researchers has developed two types of nanoparticles with reactive oxygen species scavenging ability and self-assembling redox properties. One of them, called RNP^N^, can decompose under acidic conditions; the other, called RNP^O^, can maintain its structural integrity under different pH environments and can sustain its antioxidant effect. RNP^N^ is protonated by the amino group on its side chain in an acidic environment, and the nitro radicals wrapped around the RNP^N^ core are exposed to the external environment, leading to the collapse of micelles, accompanied by a decrease in pH, so its antioxidant activity is significantly enhanced. Hence, its antioxidant activity is significantly enhanced. The experimental results showed that these two nanoparticles exhibited good bioavailability and therapeutic potential after oral administration. (Figure 7D) [72].

#### 3.2.2. Applications of Supramolecular Systems in Antimicrobial Therapy

Incorporating recognition moieties capable of selectively binding to specific polysaccharides or proteins on bacterial cell walls enables supramolecular systems to achieve targeted bacterial capture. For example, β-glucan and polysaccharide modifications on thermally reduced graphene oxide (TRGO) composites have been shown to enhance bacterial adsorption and aggregation. Upon laser irradiation, these assemblies facilitate infrared-triggered bactericidal effects. The supramolecular interactions introduced by β-glucan and polysaccharides further stabilize the system, improving bacterial binding and release performance [112].

**Figure 8:**
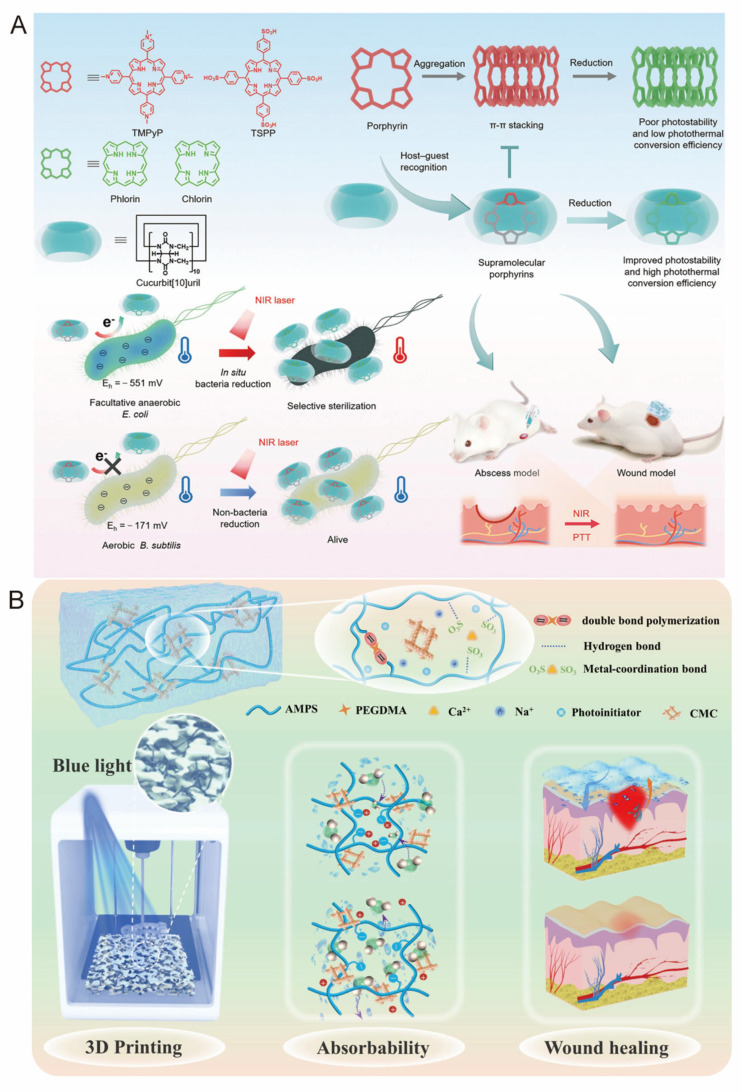
(**A**) Schematic illustration of a host–guest supramolecular porphyrin photosensitizer for in situ bacteria-responsive near-infrared photothermal therapy [73]. Copyright 2024, Wiley-VCH. (**B**) Schematic diagram of a blue light 3D-printable hydrogel with water absorption, antibacterial, and hemostatic properties for skin wound healing [75]. Copyright 2024, Elsevier.

Host–guest interactions between Cucurbit[10]uril and porphyrin have also been exploited to construct water-soluble supramolecular porphyrins with selective antimicrobial activity. Wu and colleagues developed a bacteria-responsive photothermal therapy (PTT) platform in the near-infrared region, wherein π–π stacking and hydrophobic aggregation of porphyrin molecules were effectively suppressed by Cucurbit[10]uril, leading to improved photostability and enhanced photothermal conversion efficiency. Due to the matching redox potential between facultative anaerobic *E. coli* and the porphyrin core, selective in situ reduction of the supramolecular system was achieved, enabling targeted bacterial eradication (Figure 8A) [73].

Although antimicrobial peptides (AMPs) are promising natural drugs that can effectively inhibit cervical cancer, their poor bioavailability, low tumor selectivity, and non-selective toxicity still hinder their further application in vivo. To effectively address these challenges, the Pan team developed a nanomedicine, CEC-OxbCD, which is an AMP oxidized in response to β-cyclodextrin, synthesized using a nano-self-assembly method, for the selective treatment of cervical cancer. Under the stimulation of tumor-derived reactive oxygen species (ROS), the nanodrug effectively dissociates, leading to the rapid, on-demand release of antimicrobial peptides with a 90% release rate. This supramolecular nanodrug enhances drug uptake by cells and improves drug accumulation efficiency at tumor sites. This phenomenon can be attributed to the enhanced hydrophilic stability of the supramolecular structure, thereby prolonging the circulation time in the bloodstream [74].

Antimicrobial peptide (AMP)-based supramolecular hydrogels, such as those self-assembled from C8-G(IIKK)_2_I-NH_2_ (C8G2) and C12-G(IIKK)_2_I-NH_2_ (C12G2), have demonstrated potent activity against drug-resistant pathogens. These hydrogels possess high cell compatibility and immunomodulatory potential, contributing to the healing of skin abscesses caused by methicillin-resistant *Staphylococcus aureus*. The antimicrobial mechanism involves physical disruption of bacterial membranes, leading to cytoplasmic leakage and bacterial death. In the healing phase, AMP hydrogels further recruit immune effectors and attenuate inflammation through immune response modulation [113].

Compared to traditional hydrogels, 3D-printed hydrogels offer several notable advantages. Not only do they significantly reduce production time, but they also enable the creation of materials with more complex structures [114]. Digital Light Processing (DLP) enables high-speed, high-precision 3D printing of these supramolecular hydrogels. However, it demands more extensive material preparation than direct inkjet printing [115]. Although UV-induced polymerization accelerates gel curing [116], UV exposure can damage cells and tissues [117], motivating Li et al. to develop a visible-light-activated composite initiator, combining the aqueous solution initiator 2,4,6-trimethylbenzoylphenyl phosphate triethyl ester (TPO-L) with phenyl-2,4,6-trimethylbenzoyl lithium (LAP) to successfully prepare a composite initiator. Their composite initiator (TPO-L/LAP) efficiently triggers radical polymerization under blue light (420–450 nm), eliminating the need for harmful UV irradiation [75]. Furthermore, 3D-printed gels produced with this system exhibit antibacterial activity, excellent cytocompatibility, and hemostatic function and can be customized to patient-specific wound geometries to accelerate repair (Figure 8B).

#### 3.2.3. Applications of Supramolecular Systems in Gene Therapy

Gene therapy holds significant promise for the treatment of intractable diseases by enabling precise modulation of genomic or transcriptomic targets, including both coding and non-coding RNAs. To ensure the safe and efficient delivery of therapeutic nucleic acids such as DNA, miRNA mimics, siRNA, and mRNA, both viral and non-viral delivery systems have been extensively developed [118]. Although viral vectors exhibit superior transduction efficiency, their immunogenicity, limited cargo capacity, and scalability challenges hinder their clinical translation [76].

Effective gene editing in vivo requires vectors capable of delivering editing tools to specific organs and tissues with high precision and minimal off-target effects [119]. Current strategies include virus-like particles, lipid nanoparticles, and conventional viral vectors [120]. While virus-like particles offer self-replicating features, their delivery efficiency remains suboptimal. Lipid nanoparticles are synthetically tunable, yet suffer from moderate targeting accuracy and transient expression. Viral vectors, despite high transfection efficacy, may elicit immune responses or induce cytotoxicity. Integrating supramolecular nanotechnology with signal-responsive control mechanisms presents a viable path forward to enhance delivery precision, efficiency, and safety in future applications [120].

A major challenge in tissue engineering is the formation of functional vascular networks within scaffolds [121]. Without rapid vascularization, cells embedded deep within the matrix suffer from poor nutrient diffusion and limited viability. To overcome this, genetic constructs encoding vascular endothelial growth factor (VEGF) fused with Ultrabithorax (Ubx) have been employed to promote angiogenesis within engineered scaffolds. VEGF facilitates endothelial cell proliferation, migration, and survival via activation of downstream kinases such as Akt, p38, ERK1/2, and focal adhesion kinase [122].

Co-delivery strategies integrating potent chemotherapeutic agents with gene therapy have shown synergistic effects in tumor suppression. The hydrophobic cavity of γ-CD encapsulated PTX, while pDNA formed electrostatic complexes with the cationic polymer shell, yielding positively charged nanoparticles capable of targeted delivery to folate receptor-overexpressing cancer cells. This multifunctional co-delivery system offers a promising strategy for synergistic chemo–gene therapy [29]. Although progress has been made in gene therapy, major obstacles include issues with the stability of genetic material and numerous intracellular and extracellular barriers that hinder effective gene delivery and expression [123].

## 4. Challenges and Future Directions

### 4.1. Current Challenges

Although supramolecular systems show great potential in the field of biomedical translation, they still face a series of fundamental challenges. Structurally, these systems rely on weak noncovalent interactions such as hydrogen bonds and hydrophobic interactions, making them extremely sensitive to fluctuations in temperature, pH, and ionic strength in physiological environments, and often lacking structural stability in vivo [28]. Additionally, precisely controlling their self-assembly processes—particularly regulating particle size, morphology, and monodispersity at the nanoscale—remains a major challenge [124]. Artificially designed structures often struggle to match the functional complexity and efficiency of natural biological systems (such as cell membranes) [9].

In terms of biocompatibility, certain materials may produce cytotoxic degradation products (such as long-term retained cyclodextrin derivatives), and some synthetic units require biocompatible coating modifications to avoid immunogenicity [64]. The issue of non-targeted drug release remains urgent, driving the development of stimulus-responsive carriers that can precisely respond to specific signals in the tumor microenvironment to regulate drug release kinetics [125]. Additionally, biological barriers (such as the blood–brain barrier and cell membranes) and the immune system’s rapid clearance mechanisms severely limit drug delivery efficiency [126]. Functional components (such as cell-penetrating peptides or targeting ligands) are often integrated to enhance stability and tissue specificity [7].

Another bottleneck in translational applications lies in large-scale production [27]. Existing analytical methods remain inadequate for reliably monitoring key parameters such as nanoparticle size distribution or supramolecular structural integrity. These limitations collectively hinder the clinical translation of supramolecular systems, necessitating the development of improved synthesis strategies, standardized manufacturing processes, and stringent quality control standards.

### 4.2. Future Directions

In the future, the research focus of supramolecular biomedicine will be centered on several key directions: first, developing multimodal environment-responsive carriers that can synergistically respond to tumor microenvironment characteristics (such as acidic pH, elevated reactive oxygen species, and overexpressed enzymes such as MMP-2) and exogenous stimuli (light, heat, and magnetism) to achieve integrated diagnosis and treatment [22]; second, the use of tools such as DNA origami and modular peptide scaffolds to construct customized delivery systems for personalized medicine and as high-precision spatiotemporal delivery platforms for gene editing tools like CRISPR-Cas9 [127]; and third, the design of artificial organelles and self-healing hydrogels that mimic natural structures such as mitochondria or extracellular matrix networks, for applications in metabolic disease treatment and tissue engineering [128].

At the diagnostic level, supramolecular probes integrating fluorescent or MRI reporting groups hold promise for real-time tracking of in vivo therapeutic distribution [129]; supramolecular interfaces for efficiently capturing circulating tumor cells or extracellular vesicles can enhance the accuracy of early diagnosis [130]. Advances in computational modeling and machine learning are accelerating the rational design of supramolecular materials, improving the predictive accuracy of their assembly behavior and biosafety [131]. Additionally, the use of naturally sourced biodegradable materials (such as plant fibers) will promote the development of environmentally friendly supramolecular technologies [132]. These efforts aim to build a new generation of supramolecular biomedical systems that are efficient, safe, scalable, and ecologically sustainable.

## 5. Conclusions

Supramolecular systems, defined by their dynamic reversibility and stimuli-responsiveness mediated through non-covalent interactions, have emerged as a multidisciplinary research frontier with substantial implications for biomedicine. This review has delineated major supramolecular architectures—spanning dynamic covalent and non-covalent assemblies—alongside their underlying interaction mechanisms, structural design principles, and functional applications. Dynamic covalent systems, employing reversible linkages such as disulfide, boronate ester, and hydrazone bonds, enable targeted degradation and controlled drug release within the tumor microenvironment, offering innovative strategies for cancer therapy. Meanwhile, non-covalent systems leverage interactions such as hydrogen bonding and metal coordination to support adaptive, reversible behavior. Representative host–guest complexes—including cyclodextrins, cucurbiturils, and pillararenes—as well as mechanically interlocked molecules (e.g., rotaxanes), facilitate intelligent drug delivery and molecular sensing.

Furthermore, dynamic hydrogen-bonded networks, metal–organic frameworks, and π–π stacked assemblies have demonstrated environmental responsiveness and multifunctionality, enabling applications in controlled drug release, antimicrobial treatment, and flexible biomedical devices. In diagnostics, supramolecular probes based on molecular recognition afford high sensitivity and selectivity for biomarker detection and imaging, improving the accuracy of early disease diagnosis. In therapeutic contexts, supramolecular nanocarriers, antimicrobial materials, and gene-delivery platforms leverage dynamic behavior to enhance efficacy, biocompatibility, and target specificity.

Nonetheless, significant challenges remain. Issues such as structural instability, limited control over self-assembly, immunogenicity, suboptimal delivery efficiency, and difficulties in scalable production continue to impede clinical translation. To overcome these barriers, future research must focus on the design of intelligent, stimuli-responsive carriers (e.g., pH-, enzyme-, or light-triggered systems) and personalized therapeutic platforms that integrate gene-editing technologies and tailored delivery vehicles. The incorporation of biomimetic structures, multimodal imaging modalities, and AI-driven material design is expected to facilitate precise diagnostics and treatment. Concurrently, the development of biodegradable, self-healing, and eco-friendly materials will be critical to advancing supramolecular technologies toward sustainable and clinically translatable solutions.

## Figures and Tables

**Table 1:** Classification of supramolecular systems and their biomedical applications.

Type of Supramolecular System	Interaction Mechanism	Representative Structures	Biomedical Applications	Challenges	References
Dynamic covalent supramolecules	Reversible covalent bonds	Disulfide bond, hydrazone bond, boronate ester bond	Biodegradable drug carriers, tissue engineering scaffolds	Short half-life, instability under physiological conditions	[34,35,36,37,38,39,40,41,42]
Host–guest supramolecular systems	Macrocyclic hosts, mechanically interlocked molecules	Crown ethers, cyclodextrins, pillararenes, rotaxanes	Smart drug delivery, PDT/PTT, intracellular imaging	Limited targeting efficiency, non-specific binding, biological compatibility	[43,44,45,46]
Hydrogen-bonding network systems	Hydrogen bonding	Amine–carboxyl, HOFs, multi-hydrogen-bond arrays	Biomimetic scaffolds, drug delivery	Susceptible to physiological interference, structural homogeneity issues	[47,48,49,50,51,52,53,54,55,56,57,58,59]
Metal-coordinated supramolecular systems	Coordination between metals and ligands	Ru^2+^–bipyridine, Gd^3+^–DOTA, Mg-based MOFs	Antibacterial materials, MRI contrast agents	Potential metal toxicity, limited coordination stability	[60,61,62,63,64,65]
Host–guest supramolecular systems	Interlocked molecules	β-cyclodextrin	Fluorescence imaging	-	[66]
Covalent supramolecules	Covalent bond	Imine bond	Fluorescence imaging, tumor treatment	-	[67]
Covalent supramolecules	Covalent bond	Amide bonds	PTT	-	[68]
Host–guest supramolecular systems	Interlocked molecules	Cucurucide	Contrast agent	-	[31]
Host–guest supramolecular systems	Interlocked molecules	Rotane	Fluorescent probes, imaging agents	-	[18]
Host–guest supramolecular systems	Interlocked molecules	γ-cyclodextrin	Drug delivery	-	[14]
Metal-coordinated	Coordination between metals and ligands	Ruthenium-based complexes	Drug delivery	-	[33]
Metal-coordinated	Coordination between metals and ligands	Zinc ions	Drug delivery, PDT	-	[15]
Covalent supramolecules	Covalent bond	-	Tissue engineering	-	[69]
Host–guest supramolecular systems	Interlocked molecules	β-cyclodextrin	Drug delivery	-	[70]
Covalent supramolecules	Covalent bond	Disulfide bonds	Tissue engineering	-	[71]
Covalent supramolecules	Covalent bond	PEG	Drug delivery	-	[72]
Host–guest supramolecular systems	Interlocked molecules	Cucurucide	PTT	-	[73]
Host–guest supramolecular systems	Interlocked molecules	β-cyclodextrin	Drug delivery–chemotherapy	-	[74]
Covalent supramolecules	Covalent bond	Hydrogen bond	Drug delivery–antibacterial therapy	-	[75]
Host–guest supramolecular systems	Covalent bond, interlocked molecules	Disulfide bonds, paclitaxel, γ-cyclodextrin	Drug delivery–gene therapy	-	[76]

## Data Availability

Data will be made available on request.

## References

[1] Lehn J.-M. (1988). Supramolecular chemistry—Scope and perspectives molecules, supermolecules, and molecular devices (nobel lecture). Angew. Chem. Int. Ed..

[2] Lehn J.M. (1978). Cryptates: The chemistry of macropolycyclic inclusion complexes. Acc. Chem. Res..

[3] Lehn J.-M. (1985). Supramolecular chemistry: Receptors, catalysts, and carriers. Science.

[4] Zhuang W.-R., Wang Y., Cui P.-F., Xing L., Lee J., Kim D., Jiang H.-L., Oh Y.-K. (2019). Applications of π–π stacking interactions in the design of drug-delivery systems. J. Control Release.

[5] Dong R.J., Zhou Y.F., Huang X.H., Zhu X.Y., Lu Y.F., Shen J. (2015). Functional supramolecular polymers for biomedical applications. Adv. Mater..

[6] Lundberg D.J., Brown C.M., Bobylev E.O., Oldenhuis N.J., Alfaraj Y.S., Zhao J., Kevlishvili I., Kulik H.J., Johnson J.A. (2024). Nested non-covalent interactions expand the functions of supramolecular polymer networks. Nat. Commun..

[7] Thakor A.S. (2025). The third pillar of precision medicine—Precision delivery. Medcomm.

[8] Chen Q., Sun P., Zhou J., Long T., Xiao A., Liu Z., Xu S., Lei W., Zhang R., Tian J. (2025). Renal tubular gsdme protects cisplatin nephrotoxicity by impeding ogt-stat3-s100a7a axis in male mice. Nat. Commun..

[9] Lu P., Ruan D., Huang M., Tian M., Zhu K., Gan Z., Xiao Z. (2024). Harnessing the potential of hydrogels for advanced therapeutic applications: Current achievements and future directions. Signal Transduct. Target..

[10] Liu L., Huang F., Liu J., Xiao M. (2025). Recent advances of supramolecular systems in precise cancer theranostics. Supramol. Mater..

[11] Yang X., Yang C., Zhang S., Geng H., Zhu A.X., Bernards R., Qin W., Fan J., Wang C., Gao Q. (2024). Precision treatment in advanced hepatocellular carcinoma. Cancer Cell.

[12] Joseph K., De Waal B., Jansen S.A.H., Van Der Tol J.J.B., Vantomme G., Meijer E.W. (2024). Consequences of vibrational strong coupling on supramolecular polymerization of porphyrins. J. Am. Chem. Soc..

[13] Nuthanakanti A., Srivatsan S.G. (2020). Multi-stimuli responsive heterotypic hydrogels based on nucleolipids show selective dye adsorption. Nanoscale Adv..

[14] Bai H., Wang J., Phan C.U., Chen Q., Hu X., Shao G., Zhou J., Lai L., Tang G. (2021). Cyclodextrin-based host-guest complexes loaded with regorafenib for colorectal cancer treatment. Nat. Commun..

[15] Li S., Zou Q., Li Y., Yuan C., Xing R., Yan X. (2018). Smart peptide-based supramolecular photodynamic metallo-nanodrugs designed by multicomponent coordination self-assembly. J. Am. Chem. Soc..

[16] Lai J.-C., Li L., Wang D.-P., Zhang M.-H., Mo S.-R., Wang X., Zeng K.-Y., Li C.-H., Jiang Q., You X.-Z. (2018). A rigid and healable polymer cross-linked by weak but abundant zn(ii)-carboxylate interactions. Nat. Commun..

[17] Yuan M., Chen T., Jin L., Zhang P., Xie L., Zhou S., Fan L., Wang L., Zhang C., Tang N. (2023). A carrier-free supramolecular nano-twin-drug for overcoming irinotecan-resistance and enhancing efficacy against colorectal cancer. J. Nanobiotechnol..

[18] D’orchymont F., Holland J.P. (2022). Supramolecular rotaxane-based multi-modal probes for cancer biomarker imaging. Angew. Chem. Int. Ed..

[19] Ooi Y.J., Wen Y.T., Zhu J.L., Song X., Li J. (2024). Codelivery of doxorubicin and *p53* gene by β-cyclodextrin-based supramolecular nanoparticles formed via host-guest complexation and electrostatic interaction. Biomacromolecules.

[20] Narayanan G., Shen J., Matai I., Sachdev A., Boy R., Tonelli A.E. (2022). Cyclodextrin-based nanostructures. Prog. Mater. Sci..

[21] Liu J., Wang Z., Cheng P., Zaworotko M.J., Chen Y., Zhang Z. (2022). Post-synthetic modifications of metal–organic cages. Nat. Rev. Chem..

[22] Wu X., Wang C., Wang J., Feng Y., Zhu Y., Pan Y., Yuan Y., Chen C., Cao J., Lin J. (2024). Antiadhesive, antibacterial, and anti-inflammatory sandwich-structured zif8-containing gauze for enhanced wound healing. Chem. Eng. J..

[23] Karimi M., Eslami M., Sahandi-Zangabad P., Mirab F., Farajisafiloo N., Shafaei Z., Ghosh D., Bozorgomid M., Dashkhaneh F., Hamblin M.R. (2016). Ph-sensitive stimulus-responsive nanocarriers for targeted delivery of therapeutic agents. WIREs Nanomed. Nanobiotechnol..

[24] Dheeraj S., Chandana M., Rupa M., Soumya M., Chhaya A., Deep Shikha S. (2025). Insight into the recent developments of nanoparticles in treatment of cancer and neuro-degenerative disease: A review. Curr. Top. Med. Chem..

[25] Leng J., Wang N., Chang X.L., Zhang X.P., Xu J., Yang Z.L., Qian K.L., Zheng Z.Q., Tao G.H., Jia X.D. (2025). Neodymium nitrate promotes the apoptosis of mouse liver cells via bcl2l1/caspase 3 pathway. Toxicol. Mech. Methods.

[26] Oh J.Y., Kim H.S., Palanikumar L., Go E.M., Jana B., Park S.A., Kim H.Y., Kim K., Seo J.K., Kwak S.K. (2018). Cloaking nanoparticles with protein corona shield for targeted drug delivery. Nat. Commun..

[27] Zheng Y., Baidya A., Annabi N. (2023). Molecular design of an ultra-strong tissue adhesive hydrogel with tunable multifunctionality. Bioact. Mater..

[28] Zhou Y., Wang X., Zhang D., Cui H., Tian X., Du W., Yang Z., Wan D., Qiu Z., Liu C. (2025). Precision-guided stealth missiles in biomedicine: Biological carrier-mediated nanomedicine hitchhiking strategy. Adv. Sci..

[29] Zhao F., Yin H., Li J. (2014). Supramolecular self-assembly forming a multifunctional synergistic system for targeted co-delivery of gene and drug. Biomaterials.

[30] Zhang Y., Wang X., Bai X., Li P., Su D., Zhang W., Zhang W., Tang B. (2019). Highly specific cys fluorescence probe for living mouse brain imaging via evading reaction with other biothiols. Anal. Chem..

[31] Lee H., Shahrivarkevishahi A., Lumata J.L., Luzuriaga M.A., Hagge L.M., Benjamin C.E., Brohlin O.R., Parish C.R., Firouzi H.R., Nielsen S.O. (2020). Supramolecular and biomacromolecular enhancement of metal-free magnetic resonance imaging contrast agents. Chem. Sci..

[32] Li Y., Huang F., Stang P.J., Yin S. (2024). Supramolecular coordination complexes for synergistic cancer therapy. Acc. Chem. Res..

[33] António J.P.M., Gandioso A., Nemati F., Soliman N., Vinck R., Sun F., Robert C., Burckel P., Decaudin D., Thomas C.M. (2023). Polymeric encapsulation of a ruthenium(ii) polypyridyl complex: From synthesis to in vivo studies against high-grade epithelial ovarian cancer. Chem. Sci..

[34] Zhang H., Liu T., Sun Y., Wang S., Wang W., Kuang Z., Duan M., Du T., Liu M., Wu L. (2024). Carbon-spaced tandem-disulfide bond bridge design addresses limitations of homodimer prodrug nanoassemblies: Enhancing both stability and activatability. J. Am. Chem. Soc..

[35] Sun B., Luo C., Yu H., Zhang X., Chen Q., Yang W., Wang M., Kan Q., Zhang H., Wang Y. (2018). Disulfide bond-driven oxidation- and reduction-responsive prodrug nanoassemblies for cancer therapy. Nano Lett..

[36] Ma W., Wang X., Zhang D., Mu X. (2024). Research progress of disulfide bond based tumor microenvironment targeted drug delivery system. Int. J. Nanomed..

[37] Cho S., Hwang S.Y., Oh D.X., Park J. (2021). Recent progress in self-healing polymers and hydrogels based on reversible dynamic b–o bonds: Boronic/boronate esters, borax, and benzoxaborole. J. Mater. Chem. A.

[38] Lee S.Y., Lee H., In I., Park S.Y. (2014). Ph/redox/photo responsive polymeric micelle via boronate ester and disulfide bonds with spiropyran-based photochromic polymer for cell imaging and anticancer drug delivery. Eur. Polym. J..

[39] Guo X., Shi C., Wang J., Di S., Zhou S. (2013). Ph-triggered intracellular release from actively targeting polymer micelles. Biomaterials.

[40] Shi Y., Pan X., Xu S., Zhu H., Zhao B., Sun Z., Dong R., Li N., Hou X., Yang X. (2023). Synthesis of the ph-sensitive nanoparticles based on the acylhydrazone bonds conjugated doxorubicin and studies on their in vivo anti-tumor effects. Eur. J. Med. Chem..

[41] Liu Y., Si L., Jiang Y., Jiang S., Zhang X., Li S., Chen J., Hu J. (2025). Design of ph-responsive nanomaterials based on the tumor microenvironment. Int. J. Nanomed..

[42] Hai Y., Ye H., Li Z., Zou H., Lu H., You L. (2021). Light-induced formation/scission of c–n, c–o, and c–s bonds enables switchable stability/degradability in covalent systems. J. Am. Chem. Soc..

[43] Angelova S., Nikolova V., Pereva S., Spassov T., Dudev T. (2017). A-cyclodextrin: How effectively can its hydrophobic cavity be hydrated?. J. Phys. Chem. B.

[44] Yan M., Zhou J. (2023). Pillararene-based supramolecular polymers for cancer therapy. Molecules.

[45] Xia L., Tian J., Yue T., Cao H., Chu J., Cai H., Zhang W. (2022). Pillar[5]arene-based acid-triggered supramolecular porphyrin photosensitizer for combating bacterial infections and biofilm dispersion. Adv. Healthc. Mater..

[46] Zhou J., Li J., Du X.W., Xu B. (2017). Supramolecular biofunctional materials. Biomaterials.

[47] Ren Y., Dong X. (2024). Dynamic polymeric materials via hydrogen-bond cross-linking: Effect of multiple network topologies. Prog. Polym. Sci..

[48] Annabi N., Tamayol A., Uquillas J.A., Akbari M., Bertassoni L.E., Cha C., Camci-Unal G., Dokmeci M.R., Peppas N.A., Khademhosseini A. (2014). 25th anniversary article: Rational design and applications of hydrogels in regenerative medicine. Adv. Mater..

[49] Lin Z.-J., Mahammed S.A.R., Liu T.-F., Cao R. (2022). Multifunctional porous hydrogen-bonded organic frameworks: Current status and future perspectives. ACS Cent. Sci..

[50] Pan Y., Zeng F., Luan X., He G., Qin S., Lu Q., He B., Han X., Song Y. (2025). Polyamine-depleting hydrogen-bond organic frameworks unleash dendritic cell and t cell vigor for targeted crispr/cas-assisted cancer immunotherapy. Adv. Mater..

[51] Wu J., Zeng F., Fan Z., Xuan S., Hua Z., Liu G. (2024). Hierarchical hydrogen bonds endow supramolecular polymers with high strength, toughness, and self-healing properties. Adv. Funct. Mater..

[52] Song P., Wang H. (2020). High-performance polymeric materials through hydrogen-bond cross-linking. Adv. Mater..

[53] Wang F., Yu X., Cao Z., Liu Y., Jiang X., Gu X. (2024). Synergic enhancement of hydrogel upon multi-level hydrogen bonds via macromolecular design for dual-mode electronic skin. Chem. Eng. J..

[54] Huang A., Yang H., Huang S., Chen G., Ouyang G. (2023). Hydrogen-bonded supramolecular crystal: A manual exoskeleton for bioentity. Matter.

[55] Luo J., Wang J.-W., Zhang J.-H., Lai S., Zhong D.-C. (2018). Hydrogen-bonded organic frameworks: Design, structures and potential applications. Crystengcomm.

[56] Rubio-Martinez M., Avci-Camur C., Thornton A.W., Imaz I., Maspoch D., Hill M.R. (2017). New synthetic routes towards mof production at scale. Chem. Soc. Rev..

[57] Yang J., Yang Y.-W. (2020). Metal–organic frameworks for biomedical applications. Small.

[58] Chen J., Gao Y., Shi L., Yu W., Sun Z., Zhou Y., Liu S., Mao H., Zhang D., Lu T. (2022). Phase-locked constructing dynamic supramolecular ionic conductive elastomers with superior toughness, autonomous self-healing and recyclability. Nat. Commun..

[59] Wang R., Xu T., Yang Y., Zhang M., Xie R., Cheng Y., Zhang Y. (2025). Tough polyurethane hydrogels with a multiple hydrogen-bond interlocked bicontinuous phase structure prepared by in situ water-induced microphase separation. Adv. Mater..

[60] Ranji-Burachaloo H., Gurr P.A., Dunstan D.E., Qiao G.G. (2018). Cancer treatment through nanoparticle-facilitated fenton reaction. ACS Nano.

[61] Wang Z., Wang N., Cheng S.-C., Xu K., Deng Z., Chen S., Xu Z., Xie K., Tse M.-K., Shi P. (2019). Phorbiplatin, a highly potent pt(iv) antitumor prodrug that can be controllably activated by red light. Chem.

[62] Zhou T., Zhang G., Tan C., Liu Y., Wang X.-F. (2025). Concentration gradient-induced syntheses and crystal structures of two copper(ii) coordination polymer based on phthalic acid and 2,2′-bipyridine. Molecules.

[63] Mckinlay A.C., Morris R.E., Horcajada P., Férey G., Gref R., Couvreur P., Serre C. (2010). Biomofs: Metal–organic frameworks for biological and medical applications. Angew. Chem. Int. Ed..

[64] Sukur S., Ranc V. (2025). Magnetic 2d transition-metal-based nanomaterials in biomedicine: Opportunities and challenges in cancer therapy. Materials.

[65] Cao C., Wang X., Yang N., Song X., Dong X. (2022). Recent advances of cancer chemodynamic therapy based on fenton/fenton-like chemistry. Chem. Sci..

[66] Liu Z., Zhou W., Li J., Zhang H., Dai X., Liu Y., Liu Y. (2020). High-efficiency dynamic sensing of biothiols in cancer cells with a fluorescent β-cyclodextrin supramolecular assembly. Chem. Sci..

[67] Chen J., Liang C., Song X., Yi X., Yang K., Feng L., Liu Z. (2019). Hybrid protein nano-reactors enable simultaneous increments of tumor oxygenation and iodine-131 delivery for enhanced radionuclide therapy. Small.

[68] Zeng X., Xiao Y., Lin J., Li S., Zhou H., Nong J., Xu G., Wang H., Xu F., Wu J. (2018). Near-infrared ii dye-protein complex for biomedical imaging and imaging-guided photothermal therapy. Adv. Healthc. Mater..

[69] Zengin A., Hafeez S., Habibovic P., Baker M., Van Rijt S. (2024). Extracellular matrix mimetic supramolecular hydrogels reinforced with covalent crosslinked mesoporous silica nanoparticles. J. Mater. Chem. B.

[70] Yu R., Yang Y., He J., Li M., Guo B. (2021). Novel supramolecular self-healing silk fibroin-based hydrogel via host–guest interaction as wound dressing to enhance wound healing. Chem. Eng. J..

[71] Sollenberger C.H., Qiu R., Sai H., Carrow J.K., Fyrner T., Gao Z., Palmer L.C., Stupp S.I. (2024). Boosting chondrocyte bioactivity with ultra-sulfated glycopeptide supramolecular polymers. Acta Biomater..

[72] Tang M.-D.Q., Tran N.B., Nguyen T.-H.T., Nguyen K.-U.H., Trinh N.-T., Van Vo T., Kobayashi M., Yoshitomi T., Nagasaki Y., Vong L.B. (2024). Development of oral ph-sensitive redox nanotherapeutics for gastric ulcer therapy. J. Control Release.

[73] Wu D., Du X., Xue Q., Zhou J., Ping K., Cao Y., Liu S., Zhu Q. (2024). Supramolecular porphyrin photosensitizers based on host-guest recognition for in situ bacteria-responsive near-infrared photothermal therapy. Adv. Healthc. Mater..

[74] Pan Y., Fan Z., Yu S., Xia L., Li J. (2025). Ros-responsive supramolecular antimicrobial peptides-based nanoprodrugs for cervical cancer therapy. Colloid Surf. B..

[75] He X.-C., Chen X.-N., Liu Y.-H., Zhong X., Qiang L., Wang H.-Q., Wang F.-Z., Wang J.-S., Li C.-H., Zheng P.-F. (2024). A blue light 3d printable hydrogel with water absorption, antibacterial, and hemostatic properties for skin wound healing. Chem. Eng. J..

[76] Wang H., Feng Z., Xu B. (2019). Supramolecular assemblies of peptides or nucleopeptides for gene delivery. Theranostics.

[77] Ren X., Jiang H., Cao J., Wu J., Ge F. (2025). Biobased reversible acid-sensitive colorimetric fabric sensor based on natural polyphenols and amino acid. Chem. Eng. J..

[78] Nguyen M.T.N., Nguyen T.D., Han J.-H., Lee J.S. (2024). Synthesis of pdms chain structure with introduced dynamic covalent bonding for high-performance rehealable tactile sensor application. Small Methods.

[79] Carden G.P., Martins M.L., Toleutay G., Ge S., Li B., Zhao S., Sokolov A.P. (2024). Critical role of free amine groups in the imine bonds exchange in dynamic covalent networks. Macromolecules.

[80] Liu H., Lu H.-H., Zhuang J., Thayumanavan S. (2021). Three-component dynamic covalent chemistry: From janus small molecules to functional polymers. J. Am. Chem. Soc..

[81] Liu Y., Wang L., Zhao L., Zhang Y., Li Z.-T., Huang F. (2024). Multiple hydrogen bonding driven supramolecular architectures and their biomedical applications. Chem. Soc. Rev..

[82] Jiang S., Win K.Y., Liu S., Teng C.P., Zheng Y., Han M.Y. (2013). Surface-functionalized nanoparticles for biosensing and imaging-guided therapeutics. Nanoscale.

[83] Maity S., Deb V.K., Mondal S., Chakraborty A., Pramanick K., Adhikari S. (2025). Leveraging supramolecular systems in biomedical breakthroughs. Biofactors.

[84] Wu Q., Zhou Z., Xu L., Zhong H., Xiong B., Ren T., Li Z., Yuan L., Zhang X.B. (2024). Multivalent supramolecular fluorescent probes for accurate disease imaging. Sci. Adv..

[85] Xu X.D., Lin B.B., Feng J., Wang Y., Cheng S.X., Zhang X.Z., Zhuo R.X. (2012). Biological glucose metabolism regulated peptide self-assembly as a simple visual biosensor for glucose detection. Macromol. Rapid Commun..

[86] Chagri S., Ng D.Y.W., Weil T. (2022). Designing bioresponsive nanomaterials for intracellular self-assembly. Nat. Rev. Chem..

[87] Han J., Li H., Yoon J. (2022). Activated supramolecular nano-agents: From diagnosis to imaging-guided tumor treatment. Nano Today.

[88] Hong L., Li W., Li Y., Yin S. (2023). Nanoparticle-based drug delivery systems targeting cancer cell surfaces. RSC Adv..

[89] Chen Q., Liu Z. (2016). Albumin carriers for cancer theranostics: A conventional platform with new promise. Adv. Mater..

[90] Hazarika B., Singh V.P. (2023). Macrocyclic supramolecular biomaterials in anti-cancer therapeutics. Chin. Chem. Lett..

[91] Chen Y., Huang F.H., Li Z.T., Liu Y. (2018). Controllable macrocyclic supramolecular assemblies in aqueous solution. Sci. China Chem..

[92] Nanda J., Banerjee A. (2012). Β-amino acid containing proteolitically stable dipeptide based hydrogels: Encapsulation and sustained release of some important biomolecules at physiological ph and temperature. Soft Matter.

[93] Wei X., Yu C.Y., Wei H. (2023). Application of cyclodextrin for cancer immunotherapy. Molecules.

[94] Abou-Elkacem L., Arns S., Brix G., Gremse F., Zopf D., Kiessling F., Lederle W. (2013). Regorafenib inhibits growth, angiogenesis, and metastasis in a highly aggressive, orthotopic colon cancer model. Mol. Cancer Ther..

[95] Mir O., Brodowicz T., Italiano A., Wallet J., Blay J.Y., Bertucci F., Chevreau C., Piperno-Neumann S., Bompas E., Salas S. (2016). Safety and efficacy of regorafenib in patients with advanced soft tissue sarcoma (regosarc): A randomised, double-blind, placebo-controlled, phase 2 trial. Lancet Oncol..

[96] Datta S., Misra S.K., Saha M.L., Lahiri N., Louie J., Pan D., Stang P.J. (2018). Orthogonal self-assembly of an organoplatinum(ii) metallacycle and cucurbit[8]uril that delivers curcumin to cancer cells. Proc. Natl. Acad. Sci. USA.

[97] Annane D., Bellissant E., Cavaillon J.M. (2005). Septic shock. Lancet.

[98] Fricker S.P., Slade E., Powell N.A., Vaughan O.J., Henderson G.R., Murrer B.A., Megson I.L., Bisland S.K., Flitney F.W. (1997). Ruthenium complexes as nitric oxide scavengers: A potential therapeutic approach to nitric oxide-mediated diseases. Br. J. Pharmacol..

[99] He C., Duan X., Guo N., Chan C., Poon C., Weichselbaum R.R., Lin W. (2016). Core-shell nanoscale coordination polymers combine chemotherapy and photodynamic therapy to potentiate checkpoint blockade cancer immunotherapy. Nat. Commun..

[100] Santamaria-Garcia V.J., Flores-Hernandez D.R., Contreras-Torres F.F., Cué-Sampedro R., Sánchez-Fernández J.A. (2022). Advances in the structural strategies of the self-assembly of photoresponsive supramolecular systems. Int. J. Mol. Sci..

[101] Kouwer P.H., Koepf M., Le Sage V.A., Jaspers M., Van Buul A.M., Eksteen-Akeroyd Z.H., Woltinge T., Schwartz E., Kitto H.J., Hoogenboom R. (2013). Responsive biomimetic networks from polyisocyanopeptide hydrogels. Nature.

[102] Chan G., Mooney D.J. (2008). New materials for tissue engineering: Towards greater control over the biological response. Trends Biotechnol..

[103] Lutolf M.P., Hubbell J.A. (2005). Synthetic biomaterials as instructive extracellular microenvironments for morphogenesis in tissue engineering. Nat. Biotechnol..

[104] Rijns L., Baker M.B., Dankers P.Y.W. (2024). Using chemistry to recreate the complexity of the extracellular matrix: Guidelines for supramolecular hydrogel-cell interactions. J. Am. Chem. Soc..

[105] Deng Z., Wang H., Ma P.X., Guo B. (2020). Self-healing conductive hydrogels: Preparation, properties and applications. Nanoscale.

[106] Chouhan D., Chakraborty B., Nandi S.K., Mandal B.B. (2017). Role of non-mulberry silk fibroin in deposition and regulation of extracellular matrix towards accelerated wound healing. Acta Biomater..

[107] Gou G.H., Tseng F.J., Wang S.H., Chen P.J., Shyu J.F., Weng C.F., Pan R.Y. (2020). Autologous chondrocyte implantation versus microfracture in the knee: A meta-analysis and systematic review. Arthroscopy.

[108] Niethammer T.R., Loitzsch A., Horng A., Baur-Melnyk A., Bendiks M., Gülecyüz M.F., Müller P.E., Pietschmann M.F. (2018). Graft hypertrophy after third-generation autologous chondrocyte implantation has no correlation with reduced cartilage quality: Matched-pair analysis using t2-weighted mapping. Am. J. Sports Med..

[109] Fagundes F.L., Pereira Q.C., Zarricueta M.L., Dos Santos R.D.C. (2021). Malvidin protects against and repairs peptic ulcers in mice by alleviating oxidative stress and inflammation. Nutrients.

[110] Yeomans N., Lanas A., Labenz J., Van Zanten S.V., Van Rensburg C., Rácz I., Tchernev K., Karamanolis D., Roda E., Hawkey C. (2008). Efficacy of esomeprazole (20 mg once daily) for reducing the risk of gastroduodenal ulcers associated with continuous use of low-dose aspirin. Off. J. Am. Coll. Gastroenterol.|ACG.

[111] Ünal A.T., Navruz F.Z., Korcan S.E., İnce S., Göçer E.U. (2025). Research on genotoxicity evaluation of the fungal alpha-amylase enzyme on drosophila melanogaster. Biology.

[112] Qi Z., Bharate P., Lai C.H., Ziem B., Böttcher C., Schulz A., Beckert F., Hatting B., Mülhaupt R., Seeberger P.H. (2015). Multivalency at interfaces: Supramolecular carbohydrate-functionalized graphene derivatives for bacterial capture, release, and disinfection. Nano Lett..

[113] Liu Y., Gong H., Wang Z., Yuan C., Lu J., Yan X. (2023). Treatment of superbug infection through a membrane-disruption and immune-regulation cascade effect based on supramolecular peptide hydrogels. Adv. Funct. Mater..

[114] Hou C., Gu Y., Yuan W., Zhang W., Xiu X., Lin J., Gao Y., Liu P., Chen X., Song L. (2023). Application of microfluidic chips in the simulation of the urinary system microenvironment. Mater. Today Bio.

[115] Wu D., Zhao Z., Zhang Q., Qi H.J., Fang D. (2019). Mechanics of shape distortion of dlp 3d printed structures during uv post-curing. Soft Matter.

[116] Zhang Y., Liu Y., Shu C., Shen Y., Li M., Ma N., Zhao J. (2024). 3d bioprinting of the airways and lungs for applications in tissue engineering and in vitro models. J. Tissue Eng..

[117] Jounai N., Kobiyama K., Takeshita F. (2012). Intracellular inflammatory sensors for foreign invaders and substances of self-origin. Self Nonself.

[118] Lee Y., Kim B., Lee D., Cheon S.Y., Lim S.G., Kim Y., Koo H. (2025). In vivo delivery systems for crispr genome editing: Viral and non-viral carriers. Appl. Phys. Rev..

[119] Tümmler B., Pallenberg S.T., Dittrich A.-M., Graeber S.Y., Naehrlich L., Sommerburg O., Mall M.A. (2025). Progress of personalized medicine of cystic fibrosis in the times of efficient cftr modulators. Mol. Cell. Pediatr..

[120] Raguram A., Banskota S., Liu D.R. (2022). Therapeutic in vivo delivery of gene editing agents. Cell.

[121] Meng X., Xing Y., Li J., Deng C., Li Y., Ren X., Zhang D. (2021). Rebuilding the vascular network: In vivo and in vitro approaches. Front. Cell Dev. Biol..

[122] Howell D.W., Duran C.L., Tsai S.P., Bondos S.E., Bayless K.J. (2016). Functionalization of ultrabithorax materials with vascular endothelial growth factor enhances angiogenic activity. Biomacromolecules.

[123] Xie Y., Zhou Y., Guo J., Zhang Z., Zhu Y., Goldys E.M., Deng W., Chen W. (2025). Illuminating gene delivery: Insights into the light-induced gene delivery systems with emphasis on mrna therapeutics. Mater. Today.

[124] Ma X., Zhao Y., He C., Zhou X., Qi H., Wang Y., Chen C., Wang D., Li J., Ke Y. (2021). Ordered packing of β-sheet nanofibrils into nanotubes: Multi-hierarchical assembly of designed short peptides. Nano Lett..

[125] Yan Y., Kulsoom, Sun Y., Li Y., Wang Z., Xue L., Wang F. (2025). Advancing cancer therapy: Nanomaterial-based encapsulation strategies for enhanced delivery and efficacy of curcumin. Mater. Today Bio.

[126] Ivanova A., Chalupska R., Louro A.F., Firth M., González-King Garibotti H., Hultin L., Kohl F., Lázaro-Ibáñez E., Lindgren J., Musa G. (2025). Barcoded hybrids of extracellular vesicles and lipid nanoparticles for multiplexed analysis of tissue distribution. Adv. Sci..

[127] Ferreira M., Carvalho V., Ribeiro J., Lima R.A., Teixeira S., Pinho D. (2024). Advances in microfluidic systems and numerical modeling in biomedical applications: A review. Micromachines.

[128] Zhu H., Mah Jian Qiang J., Wang C.G., Chan C.Y., Zhu Q., Ye E., Li Z., Loh X.J. (2022). Flexible polymeric patch based nanotherapeutics against non-cancer therapy. Bioact. Mater..

[129] Gostaviceanu A., Gavrilaş S., Copolovici L., Copolovici D.M. (2024). Graphene-oxide peptide-containing materials for biomedical applications. Int. J. Mol. Sci..

[130] Pandey S., Yadav P. (2025). Liquid biopsy in cancer management: Integrating diagnostics and clinical applications. Pract. Lab. Med..

[131] Oliveira C.B.P., Gomes V., Ferreira P.M.T., Martins J.A., Jervis P.J. (2022). Peptide-based supramolecular hydrogels as drug delivery agents: Recent advances. Gels.

[132] Busiak R., Masek A., Węgier A., Rylski A. (2022). Accelerated aging of epoxy biocomposites filled with cellulose. Materials.

